# Towards a rational design of solid drug nanoparticles with optimised pharmacological properties

**DOI:** 10.1002/jin2.21

**Published:** 2016-09-29

**Authors:** Marco Siccardi, Phillip Martin, Darren Smith, Paul Curley, Tom McDonald, Marco Giardiello, Neill Liptrott, Steve Rannard, Andrew Owen

**Affiliations:** ^1^ Department of Molecular and Clinical Pharmacology, Institute of Translational MedicineUniversity of LiverpoolLiverpoolL693GFUK; ^2^ Department of Chemistry, Crown StreetUniversity of LiverpoolLiverpoolL69 3BXUK

**Keywords:** Absorption, Caco‐2 cells, cellular accumulation, excipients, intestinal permeability, nanoparticles, nanotechnology, PBPK, pharmacokinetics, rational design, solid drug nanoparticle

## Abstract

Solid drug nanoparticles (SDNs) are a nanotechnology with favourable characteristics to enhance drug delivery and improve the treatment of several diseases, showing benefit for improved oral bioavailability and injectable long‐acting medicines. The physicochemical properties and composition of nanoformulations can influence the absorption, distribution, and elimination of nanoparticles; consequently, the development of nanoparticles for drug delivery should consider the potential role of nanoparticle characteristics in the definition of pharmacokinetics. The aim of this study was to investigate the pharmacological behaviour of efavirenz SDNs and the identification of optimal nanoparticle properties and composition. Seventy‐seven efavirenz SDNs were included in the analysis. Cellular accumulation was evaluated in HepG2 (hepatic) and Caco‐2 (intestinal), CEM (lymphocyte), THP1 (monocyte), and A‐THP1 (macrophage) cell lines. Apparent intestinal permeability (P_app_) was measured using a monolayer of Caco‐2 cells. The P_app_ values were used to evaluate the potential benefit on pharmacokinetics using a physiologically based pharmacokinetic model. The generated SDNs had an enhanced intestinal permeability and accumulation in different cell lines compared to the traditional formulation of efavirenz. Nanoparticle size and excipient choice influenced efavirenz apparent permeability and cellular accumulation, and this appeared to be cell line dependent. These findings represent a valuable platform for the design of SDNs, giving an empirical background for the selection of optimal nanoparticle characteristics and composition. Understanding how nanoparticle components and physicochemical properties influence pharmacological patterns will enable the rational design of SDNs with desirable pharmacokinetics.

## Introduction

Efficacy and safety of therapies are influenced by the distribution of active pharmaceutical ingredients (APIs) into tissues and organs. The pharmacokinetics of APIs can be complicated by several processes such as poor absorption, low penetration in target tissues, and high metabolism and elimination rates. Nanoformulations have been applied to tackle these issues and enhance the delivery of APIs, exploiting the specific and unique properties of sub‐micron particles. A broad spectrum of nanomedicine platforms for drug delivery has been developed using different technologies for improving pharmacokinetics, or active and passive targeting. APIs can be loaded into nanocarriers (e.g. liposomes, nanoemulsions, polymeric nanoparticles) to prevent chemical or enzymatic degradation and mediate enhanced penetration into cells, tissues, and organs (Marrache *et al*., 2013). Direct conjugation of APIs to polymers has been developed to increase circulatory times and allow the controlled release of APIs (Joralemon *et al*., 2010). Additionally, drugs can be linked to different inorganic nanoparticles, such as gold (Thakor *et al*., 2011), silver (Ong *et al*., 2013), silica (Wu *et al*., 2013), and iron (Ittrich *et al*., 2013).

Solid drug nanoparticles (SDNs) do not involve a nanocarrier system. Rather, the nanoparticle is constructed of the API, which is stabilised by polymer and/or surfactant excipients (Zhang *et al*., 2008). SDNs have proven successful with approved medicines showing benefit for improved bioavailability of poorly soluble APIs (Deschamps *et al*., 2009; McDonald *et al*., 2014), sustained release (Weir *et al*., 2013; Lyseng‐Williamson and Keating, 2002; Markowitz *et al*., 2003; Portenoy *et al*., 2002), or overcoming food effects (Deschamps *et al*., 2009; Haessler *et al*., 2008), after oral dosing. SDN formulations have also found application for long‐acting medicines where therapeutic plasma concentrations of the API are maintained for weeks or months after a single dose (van't Klooster *et al*., 2010; Baert *et al*., 2009; Spreen *et al*., 2013; Samtani *et al*., 2009).

Poor solubility and low bioavailability are continuing issues for drug development programmes in the pharmaceutical industry. Around 60% of new drug candidates have low solubility in water and biological fluids, and many have failed during development, having a detrimental effect on the success of novel therapies (Sareen *et al*., 2012; Sikarra *et al*., 2012). Many antiretroviral drugs are characterised by low aqueous solubility with poor and variable bioavailability. The absorption of APIs with poor solubility can be influenced by concomitant food intake, because of the presence of lipids increasing drug solubility (Squibb, 1999), malnutrition, and inflammatory bowel disease defining large inter‐patient variability (de Roche *et al*., 2012). For antiretrovirals, as with many other drugs, poor oral bioavailability is exacerbated in special populations, for which the intestinal anatomy and physiology can complicate absorption (Fernandez *et al*., 2011). The improvement of dissolution through nanoformulation strategies has the potential of reducing variability between patients, dose reduction, and safer treatment of special populations. We have previously described the formation of SDNs using a novel and scalable manufacturing process (Zhang *et al*., 2008; McDonald *et al*., 2014; McDonald *et al*., 2012). Moreover, efavirenz SDNs created using this approach exhibited advantages for oral bioavailability in pre‐clinical species (McDonald *et al*., 2014).

The physicochemical properties and composition of nanoformulations can impact the pharmacokinetics of nanoparticles. The size of nanoparticles has a major determining effect on their movement across biological barriers and penetration into tissues and organs. The importance of surface charge, charge density, roughness, and ligand hydrophobicity on the cellular uptake of silica nanoparticles has been recently investigated (Xue *et al*., 2013; Schrade *et al*., 2012). Additionally, commonly used excipients can impact upon the expression and activity of metabolic enzymes, suggesting that nanoparticle components have the potential to influence processes involved in absorption, distribution, metabolism, and elimination (ADME) of traditional drugs and nanoparticles (Martin *et al*., 2013). Consequently, the development of nanoparticles for drug delivery should consider the potential role of nanoparticle properties in the definition of pharmacokinetics, but these effects are likely to be composition dependent, and there are significant knowledge gaps that necessitate further investigation.

There is currently no published data showing the influence of particle properties on pharmacological characteristics of SDN formulations. Therefore, the aim of this work was to assess how nanoparticle properties relate to cellular accumulation and intestinal permeability in a library of SDNs manufactured from the same API (efavirenz) but stabilised using various excipients.

## Experimental Section

### Preparation of emulsion‐templated freeze‐dried monoliths containing efavirenz SDNs (physical science method)

^14^C‐labelled Efavirenz (specific activity: 56.7 mCi/mmol – radiochemical purity: 99.8%) was purchased from Moravek Biochemicals Inc. (Brea, CA, US). Stock solutions of efavirenz (EFV, 70 mg/mL – choloroform), polymers (22.5 mg/mL – water), and surfactants (22.5 mg/mL – water) were prepared. A solution containing a final solid mass ratio of 10% efavirenz:75% polymer and 15% surfactant in a 1:4 chloroform to water mixture was prepared. Each sample was emulsified using a Covaris S2x acoustic homogenisation system for 30 s with a duty cycle of 20, an intensity of 10 and 500 cycles/burst, in frequency sweeping mode. Following emulsification, the sample was frozen and lyophilised using a Virtis benchtop K freeze‐drier for 42 h to form a nanoparticle‐containing porous monolith. ^14^C‐labelled efavirenz was added to the stock solution to generate radioactive nanoparticles of efavirenz.

Excipients included various combinations of polymer such as polyethylene glycol 1 k (PEG1k), Pluronic F68, Pluronic F127, kollicoat protect, polyvinyl alcohol (PVA), polyvinylpyrrolidone 30 k (PVP30k), hydroxypropyl methylcellulose (HPMC), hydrolysed gelatine, and sodium carboxymethyl cellulose (NaCMC) and surfactant such as sodium alginate, sodium deoxycholate, sodium caprylate, vitamin E PEG succinate (Vit‐E PEG), Sisterna 11, Sisterna 16, sodium dodecyl sulphate (SDS), dioctyl sodium sulfosuccinate (AOT), cremophor, solutol HS, Tween 20, Tween 80, Brij58, hyamine, and cetrimonium bromide (CTAB).

### Physicochemical characterisation (physical science method)

Monoliths were dispersed in 3.5 mL of water and Zeta potential (electrical charge characteristics of the particles), particle size, and polydispersity index (heterogeneity of particle sizes) of the resulting nanoparticle dispersion were determined at a temperature of 25°C by dynamic light scattering using a Malvern Zetasizer Nano ZS equipped with a 4‐mW He–Ne, 633‐nm laser. Malvern Zetasizer software version 6.20 was used for data analysis. Measurement of the zeta potential was carried out using disposable capillary zeta cells, at pH of 6.5 and 25°C. Size, zeta potential, and polydispersity index measurements were obtained as an average of three individual measurements. Pharmacological evaluations were conducted on 77 formulations, which formed reproducible stable nanodispersions when reconstituted in water.

### Cell culture/cell maintenance (life science method)

HepG2 (hepatic) and Caco‐2 (intestinal) cells were purchased from American Type Culture Collection (ATCC; USA). Cells were maintained in Dulbecco's modified eagle's medium (DMEM; Sigma; Dorest, UK) supplemented with 10% foetal bovine serum (FBS; Bio‐Whittaker, Berkshire, UK) for HepG2 cells, and 15% sterile filtered FBS for Caco‐2 cells. THP1 (monocyte) cells were purchased from the European Collection of Cell Culture (ECACC; Porton Down, UK) and grown in RPMI‐1640 (Sigma; Dorest, UK) supplemented with 10% sterile filtered FBS. Adherent cells (HepG2 and Caco‐2) were routinely sub‐cultured every 4 days when 95% confluent. THP1 cells were sub‐cultured when a density of 1 × 10^6^ cells/mL was achieved. THP1 cells were activated to macrophage‐like cells (ATHP1) by the addition of phorbol 12‐myristate 13 acetate (PMA: Sigma; Dorest, UK) to a final concentration of 10nM in THP1 culture medium (RPMI‐1640 supplemented with 10% sterile filtered FBS). Cells were then incubated at 37°C and 5% CO_2_ for 7 days prior to use to allow differentiation from monocytes to macrophage‐like cells. Cell count and viability were determined by Trypan Blue exclusion assay. The range of passage number after receipt from ATCC or ECACC was equal to 4 – 7 for HepG2, 5 – 8 for Caco‐2, 8 – 10 for THP‐1, 8 – 10 for CEM, and 8 – 10 for ATHP‐1.

### Cellular accumulation in hepatic, intestinal, and immune cell lines (life science method)

HepG2, Caco‐2, THP1, and CEM cells were separately seeded into each well of a six‐well plate (NunclonTM, Denmark) at a density of 5 × 10^6^ cells per well in FBS supplemented DMEM (Sigma, UK) and incubated at 37°C and 5% CO_2_ for 24 h. THP1 cells were seeded at a density of 5 × 10^6^ cells per well in a six‐well plate and allowed to differentiate to ATHP1 for 7 days in the presence of 10‐nM PMA supplemented RPMI‐1640 and 10% FBS media. For HepG2, Caco‐2, and ATHP1, cells were washed with HBSS (37°C) then replaced with HBSS (37°C) containing efavirenz (10 μM/0.1 μCi ^14^C efavirenz; <0.1% DMSO) or efavirenz nanodispersions (10 μM/0.1 μCi ^14^C efavirenz). After 60‐min incubation at 37°C and 5% CO_2_, 100 μL of the extracellular solution was sampled. The remaining media was removed, and cells washed twice in ice cold HBSS. The ice cold HBSS was replaced with 500 μL of water and the plates incubated for 24 h at −20°C to lyse the cells. For THP1 and CEM cells, 5 × 10^6^ cells were separately dispensed into tubes and centrifuged at 50 ×*g* at room temperature for 1 min. The supernatant was aspirated, and cells were washed twice with HBSS (37°C). Subsequently, 600 μL of HBSS (37°C) containing efavirenz (10 μM/0.1 μCi ^14^C efavirenz; <0.1% DMSO) or efavirenz nanodispersions (10 μM/0.1 μCi ^14^C efavirenz) was added to the cells and incubated for 60 min at 37°C and 5% CO_2_. The samples were then centrifuged at 50 ×*g* for 3 min at 4°C, and 100‐μL supernatant was sampled. The cell pellet was washed twice in ice cold HBSS with centrifugation at 50 ×*g* for 3 min at 4°C. The cell pellet was lysed using 100 μL of ice cold tap water. The crude lysate was then vortexed for 30 s and transferred to a scintillation vial before incubation for 24 h at −20°C. Finally, 4 mL of Ultima Gold liquid scintillation cocktail fluid was added to all samples, and radioactivity was detected using a Perkin Elmer 3100TS scintillation counter. Average cell volumes were quantified using the Scepter 2.0 Handheld Automated Cell Counter from Merck Millipore. A cellular accumulation ratio (CAR) was then calculated as the ratio of intracellular to extracellular concentration.

### Quantification of efavirenz metabolite (life science method)

For HepG2 and ATHP1, cells were washed with HBSS (37°C) then replaced with HBSS (37°C) containing efavirenz (20 μM <0.1% DMSO) or efavirenz nanodispersions (20‐μM efavirenz). After 60‐min incubation at 37°C and 5% CO_2_, 500 μL of the extracellular solution was sampled. The remaining media was removed and cells washed twice in ice cold HBSS. The ice cold HBSS was replaced with 500 μL of trypsin and the plates incubated for 10 min 37°C and 5% CO_2_. Cell suspension was then centrifuged at 2000 rpm, 4°C for 5 min. The resulting cell pellet was resuspended in 1 mL of FBS supplemented DMEM. Subsequently, 500 μL was removed for extraction and analysis via HPLC. Caco‐2 cells were treated as above. For THP1 and CEM cells, 5 × 106 cells were separately dispensed into tubes and centrifuged at 50 ×*g* at room temperature for 1 min. The supernatant was aspirated, and cells were washed twice with HBSS (37°C). Subsequently, 1 mL of HBSS (37°C) containing efavirenz (20 μM <0.1% DMSO) or efavirenz nanodispersions (20‐μM efavirenz) was added to the cells and incubated for 60 min at 37°C and 5% CO_2_. The samples were then centrifuged at 50 ×*g* for 3 min at 4°C, and 500 μL supernatant was sampled. The cell pellet was washed twice in ice cold HBSS with centrifugation at 50 ×*g* for 3 min at 4°C. The cell pellet was resuspended in 1 mL of FBS supplemented DMEM. Subsequently, 500 μL was removed for extraction and analysis via HPLC.

Samples of media and cell suspension were treated with an excess of ACN (5:1 v/v). Samples were then thoroughly vortexed followed by centrifugation at 4000 RCF, 4°C for 10 min. The supernatant was then transferred to fresh glass vials and placed in a rotary evaporator overnight at room temperature. Samples were then reconstituted in ACN:H_2_O (20:80% V/V) and analysed via HPLC.

HPLC was performed on a Dionex system (Thermo, UK) using a Fortis C18 column. Two mobile phases were used in the chromatographic run, mobile Phase A (95% dH2O, 5% ACN, 5 mM NH4FA) and mobile phase B (90% ACN, 10% H_2_O). Chromotagraphic run was conducted using a mobile phase gradient with a flow rate of 1 mL min^−1^. At the start of the run, mobile phase A was 100% until 0.1 min when mobile phase B was increased to 92% at 0.5 min. Mobile phase B was then gradually increased to 93% over 5 min. Mobile phase B was then increased to 100% at 5.6 min which was held until 6.5 min. Mobile Phase A was then increased to 100% and held till the termination of the run at 8.55 min. Efavirenz and 8‐hydroxyefavirenz were detected using a Dionex UV detector at wavelengths 220 and 254.

### Apparent permeability (P_app_) of efavirenz across Caco‐2 cell monolayers (life science method)

Caco‐2 cells were cultured as previously described (Moss *et al*., 2011). Caco‐2 cells were seeded onto polycarbonate membrane transwell plates at a density of 5 × 10^5^ cells/cm^2^; media was replaced initially after 24 h and subsequently every 48 h. Transwell plates were cultured for 21 days, and monolayer integrity was evaluated through the quantification of the transepithelial electrical resistance (TEER). TEER values of >650 Ω cm^2^ were deemed indicative of an acceptably confluent monolayer for trancellular permeation assays. The TEER value has been measured using STX2 electrodes. One set of electrodes was placed in the apical compartment and the other in the basolateral compartment. Efavirenz (10 μM/0.1 μCi ^14^C efavirenz; <0.1% DMSO) or efavirenz nanodispersions (10 μM/0.1 μCi ^14^C efavirenz) were added to the donor chamber to quantify transport in the apical to basolateral (A > B) direction. Media was sampled on an hourly basis over 4 h. Apparent permeability coefficient (P_app_) was then determined by the amount of efavirenz transported over time using the following equation:

$$P_{\text{app}}=\;\left(dQ/dt\right)\;(1/\left(\text{Ax}C_{0}\right)$$

where A is the surface area of the transwell, C_0_ is the starting concentration of efavirenz in the donor chamber (10 μM), and (dQ/dt) is the amount per time (nmol s^−1^).

### Validation of size model by prediction of apparent permeability (P_app_) for scaled efavirenz SDNs (life science method)

The efavirenz SDNs presented within this manuscript contained 10 wt% efavirenz relative to the polymer and surfactant excipients in the dry state. As part of the translation towards clinical utility, the drug loading was pushed to 70 wt% efavirenz as previously described (McDonald *et al*., 2014). Therefore, in order to test the validity of P_app_‐prediction from particle size, the model was validated with the previously published 70 wt% efavirenz SDNs. For this, particle size of the 70 wt% SDNs (not included in the model development) was used to predict the P_app_ for these formulations. Validation was conducted by regression of predicted P_app_ against observed P_app_ and construction of Bland and Altman plots as previously described (Bland and Altman, 1986).

### Prediction of bioavailability and pharmacokinetics by physiologically‐based pharmacokinetic (PBPK) modelling (life science method)

The P_app_ values were used to evaluate the potential benefit on bioavailability and pharmacokinetics generated by the SDNs. A semi‐mechanistic physiologically based pharmacokinetic model for efavirenz was developed as previously described (McDonald *et al*., 2014; Siccardi *et al*., 2013). The model is based on the assumption that following oral administration the SDNs are not reaching the systemic circulation but are only affecting the absorption process. Once efavirenz has permeated the intestinal wall, it diffuses into the blood stream following distribution patterns similar to the traditional formulation. Efavirenz pharmacokinetics were simulated through a PBPK model developed using Berkeley Madonna (version 8.3.18, University of California, CA, USA). Experimental *in vitro* data describing the physiochemical properties, metabolism, and induction potential of efavirenz were obtained from previously published reports (Ogburn *et al*., 2010; Ward *et al*., 2003). The total hepatic clearance was calculated considering the expression of cytochrome P450, amount of microsomal protein per gram of liver, liver weight, and regional blood flows (Crewe *et al*., 2011; Ohtsuki *et al*., 2012). A population physiology model (physB) was used to generate virtual patients. Organ weights and blood flows were allometrically scaled to an individual's characteristics as previously described (Bosgra *et al*., 2012). P_app_ in Caco‐2 cells was used to derive the constant of absorption (k_a_) and oral bioavailability and then integrated in a compartmental absorption and transit model. Efavirenz distribution was simulated considering plasma to tissue ratio (Poulin and Theil, 2002), organ volumes, and blood flows. The pharmacokinetics of the conventional formulation and SDNs of efavirenz were simulated considering a 600 mg once daily dose, administered to 50 virtual subjects (20–50 years old, 0.5 proportion females) for 21 days.

### Statistical analysis (life science method)

Data were assessed for normality using a Shapiro–Wilk test. Categorical data were analysed using a Mann–Whitney test. Spearman's rank correlation was used to investigate continuous data. Univariate and stepwise‐elimination multivariate logistic regression analyses were conducted to characterise (1) the effect of chemical composition on physicochemical properties, (2) the effect of physicochemical properties on CAR and P_app_, and (3) the effect of chemical composition on CAR and P_app_.

## Results

### Physicochemical properties (physical science results)

**Figure 1:**
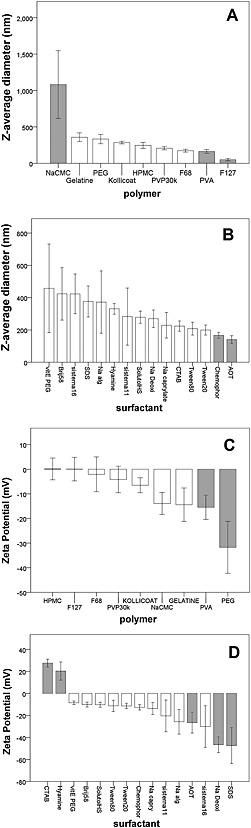
Nanoparticle size (A–B) and zeta potential (C–D) by polymers and surfactants. Bars represent mean value, and error bars represent standard error of the mean. Grey bars highlight factors significantly associated with the relevant physical property through regression analysis (*P* < 0.05).

A total of 77 efavirenz SDNs were included in the analysis. Measured *z*‐average diameter, polydispersity index and zeta‐potential (mean ± SD) were 280 ± 254 nm, 0.33 ± 0.19 and −10.7 ± 21.8 mV, respectively. A total of 65 out of 77 SDNs were characterised by negative zeta potential. The effect of SDN composition on size was investigated using multivariate linear regression. As represented in Figure 1A and B, Log_10_ *z*‐average diameter was correlated with PVA (*β* = −0.26, *p* = 0.002), F127 (*β* = −0.802, *p* = 0.0001), NaCMC (*β* = 0.542, *p* = 0.0001), AOT (*β* = −0.318, *p* = 0.02), and cremophor (*β* = −0.27, *p* = 0.001). As represented in Figure 1C and D, zeta potential was correlated with CTAB (*β* = 36.7, *p* = 0.0001), hyamine (*β* = 33.7, *p* = 0.0001), PEG 1 k (*β* = −19.2, *p* = 0.0001), SDS (*β* = −31.7, *p* = 0.001), sodium deoxycholate (*β* = −27.8, *p* = 0.002), AOT (*β* = −13.5, *p* = 0.02), and PVA (*β* = −10.4, *p* = 0.026).

### Cellular accumulation in hepatic, intestinal, and immune cell lines (life science results)

**Figure 2:**
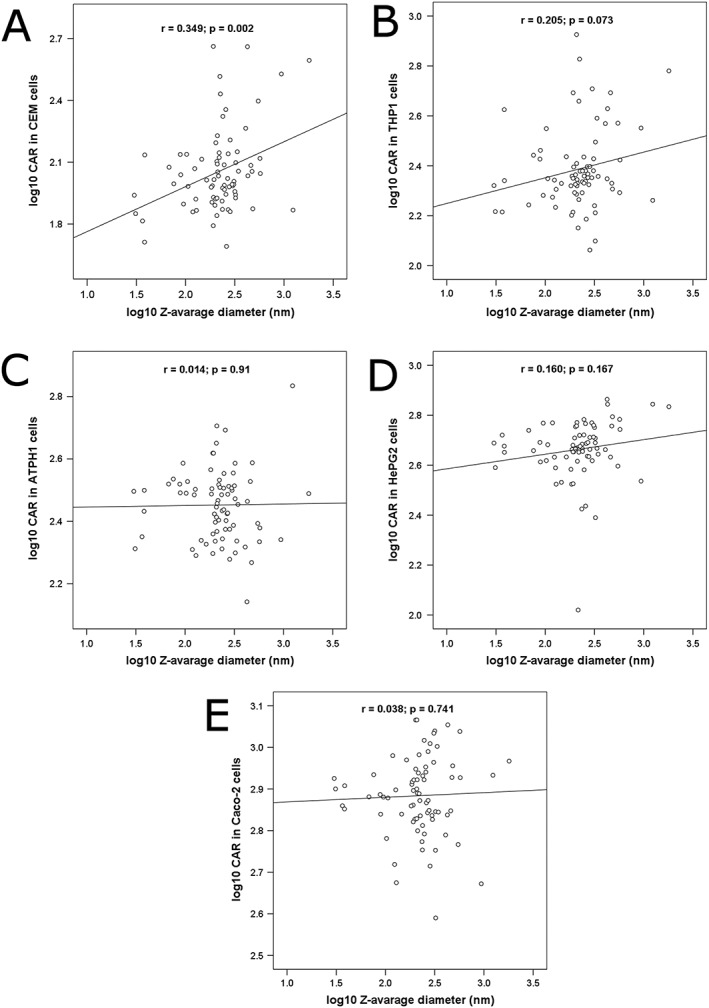
Correlation between nanoparticle diameter and CAR in CEM (A), THP1 (B), ATHP1 (C), HepG2 (D), and Caco‐2 (E) cells. *n* = 2 in duplicate were used for all cell types.

For the efavirenz SDNs (relative to aqueous efavirenz control), CAR ranged from 49 to 458 (77 for aqueous control) for CEM, 115 to 842 (202 for aqueous control) for THP1, 138 to 682 (496 for aqueous control) for ATHP1, 389 to 1163 (901 for aqueous control) for Caco‐2, and 107 to 730 for HepG2 (682 for aqueous control). The CAR of efavirenz SDNs was then compared with the accumulation of aqueous solution of efavirenz. The CAR was higher for 73% of SDNs in THP1 cells, 3% in ATHP1 cells, 88% in CEM cells, 7% in HEPG2 cells, and 21% in Caco‐2 cells. As represented in Figure 2A, log_10_ *z*‐average diameter was correlated with CAR in CEM (*r* = 0.349, *p* = 0.002) but was not significant for other cell lines (Fig. 2B–E). Zeta potential was correlated with CAR in Caco‐2 (rho = 0.258, *p* = 0.023) and HepG2 (rho = 0.230, *p* = 0.045). The results of the univariate and multivariate regression analysis for the different cell lines are summarised in Supplemental Tables 1–5. F68 (*β* = 0.14, *p* = 0.014), sodium alginate (*β* = 0.202, *p* = 0.007), sisterna 16 (*β* = 0.298, *p* = 0.004), and Tween 80 (*β* = 0.178, *p* = 0.004) were identified as associated with the accumulation in THP1. CAR in Caco‐2 cells was associated with zeta potential (*β* = 0.001, *p* = 0.023) and with PEG1K (*β* = −0.12, *p* = 0.0001). PEG1K (*β* = −0.103, *p* = 0.01), PVA (*β* = 0.072, *p* = 0.047), NaCMC (*β* = 0.197, *p* = 0.01), and Brij58 (*β* = 0.103, *p* = 0.014) in ATHP1 cells. Particle size (*β* = 0.257 *p* = 0.0001), F127 (*β* = −0.272 *p* = 0.002), and Solutol HS (*β* = −0.183 *p* = 0.015) were associated with CAR in CEM cells. For HepG2 cells, zeta potential (*β* = 0.001 *p* = 0.029), log_10_ polydispersity index (*β* = 0.113 *p* = 0.044), F68 (*β* = −0.107 *p* = 0.001), NaCMC (*β* = 0.137 *p* = 0.006), and Na deoxycholate (*β* = −0.474 *p* = 0.0001) were identified as factors associated with CAR.

### Quantification of efavirenz metabolite (life science results)

The peak height and area of the extracted samples were recorded (Supplemental Table 6) and compared to a chemical mix of efavirenz (retention time 5.4 min) and 8‐hydroxyefavirenz (retention time 4.9 min) (5000 ng mL^−1^). The table shows detectable levels of efavirenz in all cell lines in the extracted media (extracellular) and cell (intracellular) samples. In contrast, there was no detectable 8‐hydroxyefavirenz in all cell lines in the extracted media (extracellular) or cell (intracellular) samples.

### Transcellular permeability of efavirenz nanodispersions across Caco‐2 cell monolayers (life science results)

**Figure 3:**
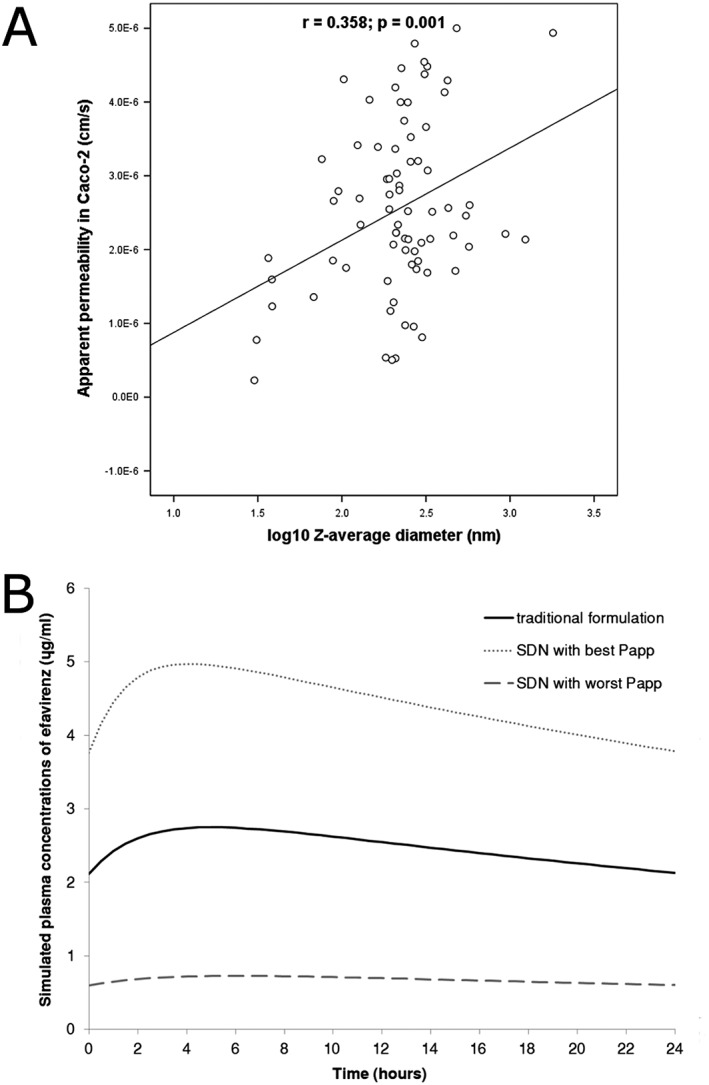
Apparent permeability of SDNs. A) Correlation between nanoparticle diameter and apparent permeability across Caco‐2 cells. B) Simulated plasma concentrations trough the PBPK model. The grey lines represent the simulated pharmacokinetics for the SDN with highest and lowest Papp; black line represents the pharmacokinetics for the traditional formulation. *n* = 2 in duplicate were used for the quantification of the P_app_.

Apparent permeability of the efavirenz SDNs ranged from 1.14 × 10^−7^ to 5.2 × 10^−6^ cm/s relative to 1.69 × 10^−6^ cm/s for the aqueous control of efavirenz, with 80.5% (62 out of 77) of the SDNs having a higher P_app_ than the efavirenz solution (<0.1% DMSO). As represented in Figure 3, a significant correlation between *z*‐average diameter and P_app_ was observed (rho = 0.34, *p* = 0.003). We have classified the SDNs in four groups based on their diameter (from 0 to 100 nm, 100–200 nm, 200–300 nm, and above 300 nm). The Papp is characterised by a significant increase for SDNs with increasing size up to 300 nm, *p* = 0.035. The mean apparent permeability of the particles characterised with >300 nm is 73% compared to the SDNs with diameter < 100 nm (*p* = 0.004) confirming the relevant influence of size in the definition of efavirenz permeability through the Caco‐2 monolayer. As summarised in Supplemental Table 7, regression analysis identified particle diameter (log_10_ *z*‐average diameter, *β* = .32, *p* = 0.001) as a predictor of P_app_. Also, nanoparticle composition appeared to be important with F68 (*β* = .24, *p* = 0.007) and PVP30k (*β* = .22, *p* = 0.0044) being significantly associated with P_app_.

### Prediction of bioavailability and pharmacokinetics by PBPK (life science results)

The prediction of plasma pharmacokinetics of the efavirenz traditional formulation was comparable to available clinical data (Vrouenraets *et al*., 2007). Simulated PK variables at steady state (mean ± SD) were C_trough_ (2203 ± 2151 ng/mL), C_max_ (2811 ± 2104 ng/mL), and AUC (59 550 ± 41 423 ng/mL h), in agreement with previous clinical PK data: C_trough_ (1752 ± 1001 ng/mL), C_max_ (4037 ± 1158 ng/mL), and AUC (57 592 ± 22 849 ng/mL h). Simulated bioavailability (mean ± SD) of the traditional formulation was 35% ± 2.6%. For context, absolute bioavailability in patients has been estimated to be around 40% (Chiappetta *et al*., 2010). As represented in Figure 3B, the simulated plasma pharmacokinetics of the SDN with best P_app_ (5.2 × 10^−6^ cm/s) resulted in an increase of 77% in C_trough_, 81% in C_max_, and 80% in AUC with an absolute bioavailability of 52% ± 3%. Conversely, the SDN with the worst P_app_ was predicted to generate a decrease of 72% in C_trough_, 76% in C_max_ and 74% in AUC with an absolute bioavailability of 7.2% ± 0.8%.

### Validation of size model by prediction of P_app_ for scaled efavirenz SDNs (life science results)

**Figure 4:**
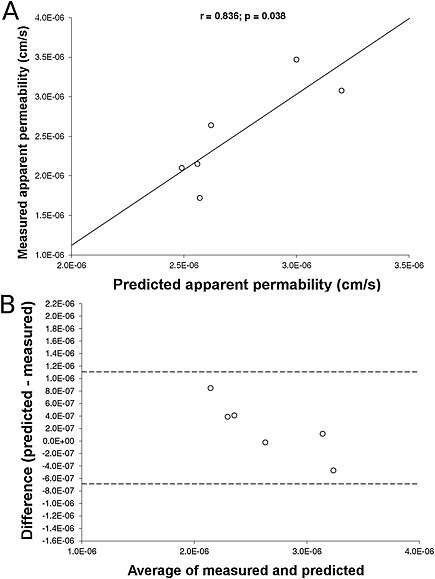
A) Correlation between predicted and measure P_app_ for the validation SDNs with 70 wt% efavirenz loading. B) Bland and Altman plot showing agreement between the predicted and measured P_app_ (within 2SD). *n* = 2 in duplicate were used for all cell types.

The P_app_‐size regression model was validated using six SDNs with 70 wt% efavirenz that were previously described and include the existing efavirenz lead formulation (McDonald *et al*., 2014). The equation describing a correlation between particle size and P_app_ was validated against the P_app_ experimentally measured for the six additional SDNs. As shown in Figure 4A, a significant correlation (*r* = 0.836, *p* = 0.038) was observed between the predicted and experimentally determined P_app_. Additionally, a Bland and Altman plot (Fig. 4B) demonstrated agreement between the predicted and measured P_app_, with all values lying between the 95% limits of agreement (±2SD).

## Discussion

This study focused on the investigation of the pharmacological behaviour of efavirenz SDNs and the identification of ideal nanoparticle properties and composition. The generated efavirenz SDNs were characterised by an improved intestinal permeability and accumulation in different cell lines compared to an aqueous solution (<0.1% DMSO) of efavirenz. The physicochemical properties of efavirenz SDNs were described and correlated with pharmacological characteristics in order to rationalise SDN optimisation for translation. The P_app_ model was validated against the previously published lead formulation and backup candidates to demonstrate the feasibility of this approach for future development. The data were important to select the current efavirenz lead formulation, progressing towards human trials, and are also of interest to inform future rational development of SDNs with favourable pharmacological features.

Cell lines used in this study were selected to represent cell populations with which nanoparticles may interact. The data should be interpreted in the context that while intestinal enterocytes are encountered by SDNs after oral administration, SDNs have not been demonstrated to enter the systemic circulation after oral or parenteral administration (Liptrott *et al*., 2014); however, other APIs are being investigated for direct intravenous administration as SDNs (Zhang *et al*., 2014). Consequently, different routes of administration may result in distinct distribution patterns because of potential increased accumulation of SDNs in tissues. Notwithstanding, Caco‐2 cells were selected to model enterocytes, HepG2 to represent hepatocytes, human acute monocytic leukaemia cells (THP1) and adapted human acute monocytic leukaemia cells (ATHP1) to model monocytes and macrophages, and human T lymphocyte cells (CEM) to represent lymphocytes. Apparent permeability through a monolayer of Caco‐2 cells is considered as a reliable model to investigate the intestinal absorption of drugs (van Breemen and Li, 2005).

Efavirenz is characterised by high membrane permeability and low aqueous solubility and classified as a BCS Class II drug (da Costa *et al*., 2012). The dissolution of this kind of API may be improved by formation of SDNs. SDNs of other APIs have been clinically demonstrated to increase intestinal permeability, but the precise mechanism for this has not been conclusively shown. The solubilisation from solid dispersions is regulated by particle size, surface area, and increased wettability. Several types of nanoparticles have been developed in recent years to improve oral absorption and/or cellular penetration through this mechanism. Improved oral bioavailability has been demonstrated by SDN formulations of other APIs (Deschamps *et al*., 2009; Weir *et al*., 2013), and other types of nanoformulation have also shown promise (Qhattal *et al*., 2011; Doh *et al*., 2013). For example, nanoemulsions of the chemopreventive agent benzyl isothiocyanate showed improved dissolution profiles, enhanced solubility, cellular accumulation, and intestinal permeability compared to traditional formulations (Qhattal *et al*., 2011). Similarly, a nanoemulsion for the antiemetic granisetron resulted in higher drug dissolution and *in vitro* permeation in Caco‐2 cells than traditional suspensions (Doh *et al*., 2013). Importantly, not all the developed efavirenz SDNs were predicted to exhibit favourable bioavailability compared to the conventional formulation. Apparent permeability through Caco‐2 cell monolayers was higher for 80.5% of the SDNs tested. Because traditionally only very few nanoparticle options are tested for an individual drug, this dataset provided a unique opportunity to probe which nanoparticle characteristics influence the observed benefits. To underscore this point, the effect of SDN formation on efavirenz pharmacokinetics was simulated through PBPK modelling. While the SDN with the highest P_app_ was predicted to result in an 80% increase in exposure, the SDN with the lowest P_app_ was predicted to result in a 74% decrease of exposure. These findings demonstrate that the arbitrary application of nanoformulation may generate unfavourable pharmacokinetic outcomes that may result in a technology being overlooked without thorough investigation. The selection of nanoparticle candidates should be based on a rational analysis of pharmacological properties to select lead formulations. Importantly, size was associated with apparent permeability across Caco‐2 cells, and this association was validated against independent SDNs containing 70 wt% efavirenz relative to polymer and surfactant excipients. The assumption that P_app_ for 70% efavirenz particles is likely to be similar to particles with equal size but lower efavirenz content is important but it should be carefully considered. This assumption could be tenuous if the surface coverage/outer layer is not characterised by the excipients and surfactants in both cases. The predicted P_app_ was in good agreement with the experimentally determined P_app_, and this approach will have utility for accelerating development of formulations with favourable properties for future APIs.

Formation of SDNs also did not guarantee benefits for cellular accumulation of efavirenz. For the cell lines ATHP1, HEPG2, and Caco‐2, the accumulation in cells was lower than the traditional formulation for the 97%, 93%, and 79% of tested SDNs, respectively. The CAR was higher for 73% of SDNs when tested in THP1 cells and 88% in CEM cells indicating preferential effects for cells in suspension compared to adherent cells, exemplified by comparing THP1 to ATHP1 cells. These data indicate that certain SDNs have the potential of enhancing drug delivery in specific cell populations while mitigating penetration to others, and this warrants further study for passive targeting applications. To further understand this, additional analysis of the factors influencing cellular accumulation was undertaken. Nanoparticle diameter was correlated with CAR only in CEM cells. Zeta potential had a positive influence on CAR in Caco‐2 and HepG2 cells. Polydispersity index was associated with higher CAR in HepG2. Also, polymers and surfactants appeared to influence cellular accumulation in different cell lines. The surfactant, F68, had a positive effect on the apparent permeability in Caco‐2 cells and cellular accumulation in THP1 while having a negative influence on the accumulation in HepG2 cells. PEG1k negatively influenced the accumulation in two cell lines, ATHP1 and Caco‐2. NaCMC had a positive effect on the CAR of ATHP1 and HepG2. Other nanoparticle components significantly influencing cellular accumulation were sodium alginate, Sisterna 16 and Tween 80 for THP1 cells, PVA and Brij58 for ATHP1, and F127 and Solutol HS for CEM cells (negative effect on accumulation). CAR in HepG2 was negatively affected by Na deoxycholate and F68. Additionally, apparent permeability in Caco‐2 cells was positively associated with PVP30k and F68. Several nanoparticle properties such as composition, surface chemistry, size, and shape can have an effect on pharmacokinetics, influencing uptake and transport in tissues across different technological platforms (Moss and Siccardi, 2014). The role of structure and surface functionality on the definition of ADME properties of dendrimers have been recently characterised (Kaminskas *et al*., 2011). Size was identified as a predictor of plasma concentrations and biodistribution for gold nanoparticle, with small nanoparticles (4 or 13 nm) detectable for up to 7 days in blood, whereas large (100 nm) nanoparticles were completely cleared in 24 h (Cho *et al*., 2010). Additionally, the elimination processes of polymeric nanoparticles are influenced by numerous variables including choice of polymer, size, charge, and the use of active targeting strategies. A correlation between charge, size, and clearance has been observed, with smaller nanoparticle having a faster clearance (Alexis *et al*., 2008). Our findings may indicate that the mechanisms regulating SND accumulation may differ between cell types. Because the excipients used influenced the particle properties, it was not possible to delineate one from the other; however, collectively, these data provide a platform from which to select excipients forming nanoparticles with physical properties for favourable pharmacological behaviour.

The presented data indicate that size can be tuned through appropriate selection of excipients. PVA, F127, AOT, and cremophor were predictors of smaller nanoparticle diameter, and NaCMC was correlated with increased diameter when using this processing technique. The appropriate selection of polymers and surfactant could be an important factor to influence nanoparticle size. Nanoparticle surface charge was also influenced by the chemical composition, with PVA and PEG1k associated with a negative zeta potential and CTAB and hyamine correlating with a positive surface charge as would be expected as CTAB and hyamine are industrial cationic surfactants. Previous studies have also highlighted the importance of nanoparticle composition, characteristics, and geometry in the definition of their pharmacokinetics (Xue *et al*., 2013; Schrade *et al*., 2012). Phagocytosis and cellular accumulation in macrophages have been shown to be inhibited by a combination of elongated shape and PEGylation (Mathaes *et al*., 2014). For non‐phagocytic cells, the uptake has been shown to be influenced by size and charge with variability between different cell populations and ambiguous results regarding shape (Kettler *et al*., 2014). In our study, size was weakly correlated with cellular accumulation for CEM cells which are not characterised by phagocytic uptake and are not adherent. Consequently, it could be hypothesised that specific mechanisms only present in cells with similar characteristics can define these effects. However, a universal correlation between nanoparticle properties and distribution is unlikely considering the broad variability in available nanotechnologies and the complex interplay between nanoparticle characteristics and pharmacokinetics. Also, it remains to be seen whether similar correlations are observed across APIs or whether effects are API specific. Additionally, the selected methodologies are based on static cell culture and potentially nanoparticle dynamics of uptake and degree of accumulation can be influenced by flow of biological fluids such as intestinal buffer or blood and plasma.

Numerous mechanisms may underpin the higher accumulation and permeability of SDNs. Nanoparticles can be taken up into cells following the interaction with specific receptors triggering endocytosis, pinocytosis, or phagocytosis. The cellular entry of polystyrene beads has been investigated in Hela and MDCK cell lines, highlighting that nanoparticle internalisation depends upon clathrin‐mediated endocytosis and direct permeabilisation of the plasma membrane (Smith *et al*., 2012). In other studies, the uptake through phagocytosis by macrophages was clarified for nanoparticles such as polylactic acid nanoparticles (Li *et al*., 2013). In the intestine, uptake of nanoparticles has been described to happen through paracellular pathways and transcellularly by enterocytes and Peyer's patches via M‐cells (Jia, 2005). However, to the best of our knowledge such experiments have not been conducted for untargeted SDNs. Nonetheless, different processes are likely to interplay to govern nanoparticle penetration into cells.

The activity of transporters has a relevant role in mediating drug movement through biological membranes and intracellular penetration. Nanoparticles may be able to circumvent the action of transporters by protecting the drug from efflux and increasing cellular accumulation or crossing biological membranes. In this regard, antiretrovirals, cardiovascular drugs, anti‐cancer agents, antibiotics, and a plethora of other APIs are substrates of efflux transporters such as ABCB1 and ABCCs (Lee *et al*., 1998; Togami *et al*., 2014; Wessler *et al*., 2013). Transporters can determine high inter‐patient variability in drug distribution, considering multiple genetic and environmental factors influencing their expression and activity. Additionally, drug–drug and food–drug interactions are a relevant concern in several treatments and are often mediated by efflux and influx transporters. Transporters such as ABCB1 have frequently overlapping substrate and inhibitor specificities with CYP3A4, and therefore drug–drug interactions can result in complex pharmacokinetics. The possibility of countering the effect of transporters on drug distribution is interesting, and excipients included in SDN formulation have the potential to alter the activity of drug metabolism enzymes *in vitro* (Martin et al., 2013). The influence of polymers and surfactants on transporters and other mechanisms of drug/nanoparticle accumulation might explain the broad variability observed between different cell lines in the current study. However, the effects of many nanoparticle excipients on the activity and expression levels of metabolism enzymes and drug transporters *in vivo* are not fully understood.

In summary, nanoparticle size and excipient choice influenced apparent permeability and cellular accumulation, and this appeared to be cell line dependent. These data represent a useful platform for the optimisation of SDNs, giving an empirical background for the selection of composition and physical characteristics. Understanding how excipients and nanoparticle physical properties influence biological phenotypes across different APIs will enable future rational design of SDNs with desirable pharmacological properties. The analysis of property–distribution relationships is essential to inform the design of nanoparticle with optimal pharmacokinetics and enhanced drug distribution.

## Competing Interest Statement

None.

## Supporting information

Supporting info item
