# Supplementary material for: Towards a rational design of solid drug nanoparticles with optimised pharmacological properties
Source: J Interdiscip Nanomed. 2016 Sep 29;1(3):110–23. doi: 10.1002/jin2.21 (PMC5054800; doi:10.1002/jin2.21)
Supplement: Supplementary file 1 — Supporting info item [file JIN2-1-110-s001.docx]

**Supplemental Information**

| **Supplemental Table 1A.** Univariate and multivariate regression analysis for the identification of SDN physicochemical properties (as measured by dynamic light scattering) influencing CAR in THP1 cells | | | | |
| --- | --- | --- | --- | --- |
|  | **Univariate** | | **Multivariate** | |
|  | **β (95% CI)** | **P value** | **β (95% CI)** | **P value** |
| log_10_ z-average diameter | 0.1 (-0.01 to 0.215) | .073 | 0.103 (-0.102 to 0.215) | 0.073 |
| Rank zeta potential | n.s. | 0.86 | - | - |
| log_10_ polydispersity index | 0.138 (-0.014 to 0.290) | 0.074 | n.s. | 0.102 |

| **Supplemental Table 1B.** Univariate and multivariate regression analysis for the identification of SDN composition influencing CAR in THP1 cells. | | | | |
| --- | --- | --- | --- | --- |
|  | **Univariate** | | **Multivariate** | |
|  | **β (95% CI)** | **P value** | **β (95% CI)** | **P value** |
| PEG 1K | n.s. | 0.89 | - | - |
| **F68** | **0.137 (0.013 to 0.261)** | **0.031** | **0.14 (0.03 to 0.253)** | **0.014** |
| F127 | n.s. | 0.42 | - | - |
| Kollicoat | n.s. | 0.63 | - | - |
| PVA | n.s. | 0.52 | - | - |
| PVP 30k | n.s. | 0.35 | - | - |
| HPMC | n.s. | 0.5 | - | - |
| Hydrolysed gelatine | n.s. | 0.32 | - | - |
| NaCMC | n.s. | 0.46 | - | - |
| **Na Alginate** | **0.014 (0.003 to 0.024)** | **0.013** | **0.202 (0.06 to 0.347)** | **0.007** |
| Na Deoxycholate | n.s. | 0.58 | - | - |
| Na Caprylate | n.s. | 0.68 | - | - |
| Vit-E PEG | n.s. | 0.49 | - | - |
| Sisterna 11 | n.s. | 0.23 | - | - |
| **Sisterna 16** | **0.26 (0.036 to 0.48)** | **0.023** | **0.298 (0.098 to 0.498)** | **0.004** |
| SDS | n.s. | 0.60 | - | - |
| AOT | n.s. | 0.63 | - | - |
| Chremophor | 0.079 (-0.2 to 0.04) | 0.19 | n.s. | 0.977 |
| Solutol HS | n.s. | 0.31 | - | - |
| Tween 20 | n.s. | 0.73 | - | - |
| **Tween 80** | **0.144 (0.011 to 0.28)** | **0.035** | **0.178 (0.058 to 0.253)** | **0.004** |
| BRij 58 | n.s. | 0.34 | - | - |
| Hyamine | n.s. | 0.38 | - | - |
| CTAB | n.s. | 0.68 | - | - |

| **Supplemental Table 2A.** Univariate and multivariate regression analysis for the identification of SDN physicochemical properties (as measured by dynamic light scattering) influencing CAR in Caco-2 cells. | | | | |
| --- | --- | --- | --- | --- |
|  | **Univariate** | | **Multivariate** | |
|  | **β (95% CI)** | **P value** | **β (95% CI)** | **P value** |
| log_10_ z-average diameter | n.s. | .741 | **-** | **-** |
| **Rank zeta potential** | **0.001 (0 to 0.002)** | **0.023** | **0.001 (0 to 0.002)** | **0.023** |
| log_10_ polydispersity index | n.s. | 0.588 | **-** | **-** |

| **Supplemental Table 2B.** Univariate and multivariate regression analysis for the identification of SDN composition influencing CAR in Caco-2 cells | | | | |
| --- | --- | --- | --- | --- |
|  | **Univariate** | | **Multivariate** | |
|  | **β (95% CI)** | **P value** | **β (95% CI)** | **P value** |
| **PEG 1K** | **-0.135 (-0.185 to -0.084)** | **0.0001** | **- 0.12 (-0.171 to -0.069)** | **0.0001** |
| F68 | n.s. | 0.268 | **-** | **-** |
| F127 | n.s. | 0.631 | - | - |
| Kollicoat | 0.047 (-0.007 to 0.102) | 0.088 | n.s. | 0.917 |
| PVA | n.s. | 0.944 | - | - |
| PVP 30k | n.s. | 0.857 | - | - |
| HPMC | n.s. | 0.230 | - | - |
| Hydrolysed gelatine | 0.067 (0.022 to 0.132) | 0.044 | 0.055 (-0.002 to 0.113) | 0.060 |
| NaCMC | n.s. | 0.287 | - | - |
| Na Alginate | -0.068 (-0.163 to 0.028) | 0.162 | n.s. | 0.989 |
| Na Deoxycholate | 0.123 (-0.255 to 0.009) | 0.068 | -0.102 (-0.219 to 0.015) | 0.086 |
| Na Caprylate | n.s. | 0.390 | - | - |
| Vit-E PEG | n.s. | 0.331 | - | - |
| Sisterna 11 | n.s. | 0.753 | - | - |
| Sisterna 16 | -0.089 (-0.233 to 0.044) | 0.186 | n.s. | 0.973 |
| SDS | n.s. | 0.757 | **-** | **-** |
| AOT | n.s. | 0.648 | **-** | **-** |
| Chremophor | n.s. | 0.618 | **-** | **-** |
| Solutol HS | n.s. | 0.779 | **-** | **-** |
| Tween 20 | n.s. | 0.813 | **-** | **-** |
| Tween 80 | n.s. | 0.256 | **-** | **-** |
| BRij 58 | n.s. | 0.238 | **-** | **-** |
| Hyamine | n.s. | 0.955 | **-** | **-** |
| CTAB | n.s. | 0.268 | **-** | **-** |

| **Supplemental Table 3A.** Univariate and multivariate regression analysis for the identification of SDN physicochemical properties (as measured by dynamic light scattering) influencing CAR in ATHP1 cells. | | | | |
| --- | --- | --- | --- | --- |
|  | **Univariate** | | **Multivariate** | |
|  | **β (95% CI)** | **P value** | **β (95% CI)** | **P value** |
| log_10_ z-average diameter | n.s. | 0.906 | - | - |
| Rank zeta potential | n.s. | 0.66 | - | - |
| log_10_ polydispersity index | 0.094 (-0.014 to 0.202) | 0.086 | 0.094 (-0.014 to 0.202) | 0.086 |

| **Supplemental Table 3B.** Univariate and multivariate regression analysis for the identification of SDN composition influencing CAR in ATHP1 cells. | | | | |
| --- | --- | --- | --- | --- |
|  | **Univariate** | | **Multivariate** | |
|  | **β (95% CI)** | **P value** | **β (95% CI)** | **P value** |
| **PEG 1K** | **-0.12 (-0.19 to -0.54)** | **0.001** | **-0.103 (-0.163 to -0.043)** | **0.01** |
| F68 | n.s. | 0.57 | - | - |
| F127 | -0.077 (-0.182 to 0.028) | 0.147 | - | 0.96 |
| Kollicoat | n.s. | 0.97 | - | - |
| **PVA** | **0.072 (-0.012 to 0.157)** | **0.09** | **0.072 (0.001 to 0.143)** | **0.047** |
| PVP 30k | n.s. | 0.25 | - | - |
| HPMC | n.s. | 0.97 | - | - |
| Hydrolysed gelatine | n.s. | 0.61 | - | - |
| **NaCMC** | **0.233 (0.11 to 0.357)** | **0.0001** | **0.197 (0.084 to 0.31)** | **0.01** |
| Na Alginate | n.s. | 0.78 | - | - |
| Na Deoxycholate | n.s. | 0.39 | - | - |
| Na Caprylate | n.s. | 0.98 | - | - |
| Vit-E PEG | n.s. | 0.96 | - | - |
| Sisterna 11 | n.s. | 0.35 | - | - |
| Sisterna 16 | n.s. | 0.94 | - | - |
| SDS | n.s. | 0.24 | - | - |
| AOT | n.s. | 0.79 | - | - |
| Chremophor | n.s. | 0.99 | - | - |
| Solutol HS | n.s. | 0.77 | - | - |
| Tween 20 | n.s. | 0.96 | - | - |
| Tween 80 | n.s. | 0.97 | - | - |
| **BRij 58** | **0.12 (0.027 to 0.214)** | **0.012** | **0.103 (0.021 to 0.184)** | **0.014** |
| Hyamine | -0.065 (-0.155 to 0.024) | 0.15 | - | 0.99 |
| CTAB | n.s. | 0.98 | - | - |

| **Supplemental Table 4A.** Univariate and multivariate regression analysis for the identification of SDN physicochemical properties influencing CAR in CEM cells. | | | | |
| --- | --- | --- | --- | --- |
|  | **Univariate** | | **Multivariate** | |
|  | **β (95% CI)** | **P value** | **β (95% CI)** | **P value** |
| **log_10_ z-average diameter** | **0.218 (0.083 to 0.35)** | **0.002** | **0.218 (0.083 to 0.352)** | **0.002** |
| Rank zeta potential | -0.001 (0.003 to 0.010) | 0.179 | - | 0.99 |
| log_10_ polydispersity index | n.s. | 0.31 | - | - |

| **Supplemental Table 4B.** Univariate and multivariate regression analysis for the identification of SDN composition influencing CAR in CEM cells. | | | | |
| --- | --- | --- | --- | --- |
|  | **Univariate** | | **Multivariate** | |
|  | **β (95% CI)** | **P value** | **β (95% CI)** | **P value** |
| PEG 1K | 0.092 (-0.033 to 0.216) | 0.148 | - | 0.94 |
| F68 | n.s. | 0.86 | - | 0.97 |
| **F127** | **-0.234 (-0.412 to -0.055)** | **0.011** | **-0.272 (-0.444 to -0.099)** | **0.002** |
| Kollicoat | -0.077 (-0.195 to 0.041) | 0.20 | -0.103 (-0.213 to -0.007) | 0.065 |
| PVA | n.s. | 0.66 | - | - |
| PVP 30k | n.s. | 0.61 | - | - |
| HPMC | n.s. | 0.92 | - | - |
| Hydrolysed gelatine | n.s. | 0.33 | - | - |
| NaCMC | n.s. | 0.25 | - | - |
| Na Alginate | n.s. | 0.27 | - | - |
| Na Deoxycholate | n.s. | 0.94 | - | - |
| Na Caprylate | n.s. | 0.99 | - | - |
| Vit-E PEG | n.s. | 0.60 | - | - |
| Sisterna 11 | n.s. | 0.89 | - | - |
| Sisterna 16 | n.s. | 0.33 | - | - |
| SDS | n.s. | 0.67 | - | - |
| AOT | n.s. | 0.47 | - | - |
| Chremophor | n.s. | 0.45 | - | - |
| **Solutol HS** | **-0.159 (-0.314 to -0.003)** | **0.045** | **-0.183 (-0.33 to -0.036)** | **0.015** |
| Tween 20 | n.s. | 0.28 | - | - |
| Tween 80 | n.s. | 0.55 | - | - |
| BRij 58 | n.s. | 0.31 | - | - |
| Hyamine | n.s. | 0.98 | - | - |
| CTAB | n.s. | 0.64 | - | - |

| **Supplemental Table 5A.** Univariate and multivariate regression analysis for the identification of SDN physicochemical properties influencing CAR in HepG2 cells. | | | | |
| --- | --- | --- | --- | --- |
|  | **Univariate** | | **Multivariate** | |
|  | **β (95% CI)** | **P value** | **β (95% CI)** | **P value** |
| log_10_ z-average diameter | 0.058 (-0.25 to 0.142 | 0.167 | - | 0.98 |
| **Rank zeta potential** | **0.001 (0 to 0.002)** | **0.045** | **0.001 (0 to 0.002)** | **0.029** |
| **log_10_ polydispersity index** | **0.104 (-0.008 to 0.217)** | **0.069** | **0.113 (0.003 to 0.223)** | **0.044** |

| **Supplemental Table 5B.** Univariate and multivariate regression analysis for the identification of SDN composition influencing CAR in HepG2 cells. | | | | |
| --- | --- | --- | --- | --- |
|  | **Univariate** | | **Multivariate** | |
|  | **β (95% CI)** | **P value** | **β (95% CI)** | **P value** |
| PEG 1K | -0.049 (-0.122 to 0.025) | 0.191 | - | 0.951 |
| **F68** | **-0.09 (-0.181 to 0.002)** | **0.055** | **-0.098 (-0.163 to -0.032)** | **0.004** |
| F127 | n.s. | 0.62 | - | - |
| Kollicoat | n.s. | 0.41 | - | - |
| PVA | n.s. | 0.58 | - | - |
| PVP 30k | n.s. | 0.38 | - | - |
| HPMC | n.s. | 0.24 | - | - |
| Hydrolysed gelatine | n.s. | 0.45 | - | - |
| **NaCMC** | **0.16 (0.025 to 0.294)** | **0.021** | **0.137(-0.04 to 0.235)** | **0.006** |
| Na Alginate | n.s. | 0.5 | - | - |
| **Na Deoxycholate** | **- 0.47 (-0.6 to -0.34)** | **0.0001** | **-0.474(-0.593 to -0.356)** | **0.0001** |
| Na Caprylate | n.s. | 0.99 | - | - |
| Vit-E PEG | n.s. | 0.67 | - | - |
| Sisterna 11 | n.s. | 0.94 | - | - |
| Sisterna 16 | n.s. | 0.5 | - | - |
| SDS | n.s. | 0.65 | - | - |
| AOT | n.s. | 0.95 | - | - |
| Chremophor | n.s. | 0.95 | - | - |
| Solutol HS | n.s. | 0.73 | - | - |
| Tween 20 | n.s. | 0.53 | - | - |
| Tween 80 | n.s. | 0.5 | - | - |
| BRij 58 | 0.073 (-0.026 to 0.172) | 0.147 | - | 0.955 |
| Hyamine | n.s. | 0.90 | - | - |
| CTAB | n.s. | 0.24 | - | - |

**Supplemental Table 6.** The peak height and peak area for efavirenz and 8-hydroxyefavirenz for blank media, chemical mix, the intracellular samples and extracellular samples.

|  |  | **EFV** | | **8-hydroxyefavirenz** | |
| --- | --- | --- | --- | --- | --- |
|  | **Condition** | **Peak Height** | **Peak Area** | **Peak Height** | **Peak Area** |
|  | Extracted Media (blank) | 0.26 | 2.37 | 0.20 | 1.23 |
|  | Chemical Mix | 9.87 | 150.45 | 8.61 | 117.27 |
| Intracellular | HEPG-2 | 8.95 | 115.10 | 0.35 | 1.42 |
|  | CACO-2 | 5.42 | 92.57 | 0.13 | 0.74 |
|  | THP1 | 6.79 | 85.31 | 0.14 | 0.79 |
|  | A THP1 | 6.32 | 82.95 | 0.24 | 1.18 |
|  | CEM | 5.96 | 71.01 | 0.21 | 0.93 |
| Extracellular | HEPG-2 | 9.24 | 106.88 | 0.06 | 0.55 |
|  | CACO-2 | 4.43 | 75.01 | 0.22 | 1.38 |
|  | THP1 | 14.29 | 207.46 | 0.13 | 0.87 |
|  | A THP1 | 14.40 | 201.35 | 0.12 | 0.73 |
|  | CEM | 16.27 | 193.99 | 0.32 | 1.44 |

| **Supplemental Table 7A.** Univariate and multivariate regression for identification of SDN characteristics (as measured by dynamic light scattering) influencing P_app_ in Caco-2 cells. | | | | |
| --- | --- | --- | --- | --- |
|  | **Univariate** | | **Multivariate** | |
|  | **β (95% CI)** | **P value** | **β (95% CI)** | **P value** |
| **log_10_ z-average diameter** | **0.28 (0.11 to 0.45)** | **.001** | **0.32 (0.16 to 0.48)** | **.0001** |
| Rank zeta potential | n.s. | 0.84 | - | - |
| log10 polydispersity index | n.s. | 0.38 | - | - |

| **Supplemental Table 7B.** Univariate and multivariate regression analysis for the identification of SDN composition influencing P_app_ in Caco-2 cells. | | | | |
| --- | --- | --- | --- | --- |
|  | **Univariate** | | **Multivariate** | |
|  | **β (95% CI)** | **P value** | **β (95% CI)** | **P value** |
| PEG 1K | n.s. | 0.96 | - | - |
| **F68** | **0.174 (-0.025 to 0.374)** | **0.085** | **0.24 (0.069 to 0.426)** | **0.007** |
| F127 | -0.445 (-.69 to -.20) | 0.0004 | n.s. | 0.196 |
| Kollicoat | n.s. | .83 | - | - |
| PVA | -0.197 (-.383 to -0.01) | .038 | n.s. | 0.387 |
| **PVP 30k** | **0.182 (0.013 to 0.35)** | **0.035** | **0.22 (0.07 to 0.376)** | **0.004** |
| HPMC | n.s. | 0.49 | - | - |
| Hydrolysed gelatine | n.s. | 0.26 | - | - |
| NaCMC | n.s. | 0.48 | - | - |
| Na Alginate | n.s. | 0.91 | - | - |
| Na Deoxycholate | n.s. | 0.68 | - | - |
| Na Caprylate | n.s. | 0.67 | - | - |
| Vit-E PEG | n.s. | 0.88 | - | - |
| Sisterna 11 | n.s. | 0.74 | - | - |
| Sisterna 16 | n.s. | 0.257 | - | - |
| SDS | n.s. | 0.58 | - | - |
| AOT | n.s. | 0.52 | - | - |
| Chremophor | n.s. | 0.78 | - | - |
| Solutol HS | n.s. | 0.61 | - | - |
| Tween 20 | n.s. | 0.53 | - | - |
| Tween 80 | n.s. | 0.79 | - | - |
| BRij 58 | 0.16 (-0.5 to 0.38) | 0.131 | n.s. | 0.223 |
| Hyamine | n.s. | 0.37 | - | - |
| CTAB | n.s. | 0.994 | - | - |
